# GDP-L-fucose is required for boundary definition in plants

**DOI:** 10.1093/jxb/erx402

**Published:** 2017-11-25

**Authors:** Beatriz Gonçalves, Aude Maugarny-Calès, Bernard Adroher, Millán Cortizo, Nero Borrega, Thomas Blein, Alice Hasson, Emilie Gineau, Grégory Mouille, Patrick Laufs, Nicolas Arnaud

**Affiliations:** Institut Jean-Pierre Bourgin, INRA, AgroParisTech, CNRS, Université Paris-Saclay, France

**Keywords:** Arabidopsis, boundaries, CUP-SHAPED COTYLEDON 2, development, GDP-L-fucose, leaf

## Abstract

The CUP-SHAPED COTYLEDON (CUC) transcription factors control plant boundary formation, thus allowing the emergence of novel growth axes. While the developmental roles of the *CUC* genes in different organs and across species are well characterized, upstream and downstream events that contribute to their function are still poorly understood. To identify new players in this network, we performed a suppressor screen of *CUC2g-m4*, a line overexpressing *CUC2* that has highly serrated leaves. We identified a mutation that simplifies leaf shape and affects *MURUS1* (*MUR1*), which is responsible for GDP-L-fucose production. Using detailed morphometric analysis, we show that GDP-L-fucose has an essential role in leaf shape acquisition by sustaining differential growth at the leaf margins. Accordingly, reduced *CUC2* expression levels are observed in *mur1* leaves. Furthermore, genetic analyses reveal a conserved role for GDP-L-fucose in different developmental contexts where it contributes to organ separation in the same pathway as CUC2. Taken together, our results reveal that GDP-L-fucose is necessary for proper establishment of boundary domains in various developmental contexts.

## Teaser

Mutation of *MURUS1* suppresses the highly serrated leaf phenotype that results from overexpression of CUP-SHAPED COTYLEDON 2 in Arabidopsis, revealing that GDP-L-fucose is involved in defining tissue boundaries during plant development.

## Introduction

In multicellular organisms, the development of distinct organs often requires the establishment of boundaries to individualize functional units. While physically separating organs, boundaries also act as organizing centers to control cell fate in neighboring tissues. Thus, formation and maintenance of boundaries is crucial to guide the establishment of reproducible developmental patterns (Dahmann *et al.*, 2011).

Several transcriptional regulators are involved in the development of boundaries in plants, including the CUP-SHAPED COTYLEDON (CUC) transcription factors (reviewed in Žádníková and Simon, 2014; Hepworth and Pautot, 2015; Maugarny *et al.*, 2015). They belong to the NAC (NO APICAL MERISTEM/ATAF1-2/CUC2) family, an evolutionarily conserved family of plant transcription factors notably involved in developmental processes and stress responses (Nuruzzaman *et al.*, 2013). As players involved in boundary definition, CUC transcription factors are major regulators of plant architecture, controlling both shoot meristem maintenance (Aida *et al.*, 1999) and correct organ separation in various developmental contexts (Aida *et al.*, 1997; Burian *et al.*, 2015; Gonçalves *et al.*, 2015). In addition, *CUC* genes are master regulators of leaf shape through their roles in leaf margin development (Hasson *et al.*, 2011; Nikovics *et al.*, 2006). Detailed analysis of leaf morphogenesis has revealed the complex morphological effects of CUC activity: they repress growth locally while promoting growth in adjacent regions through a non-cell-autonomous mechanism involving auxin (Bilsborough *et al.*, 2011; Biot *et al.*, 2016; Blein *et al.*, 2008; Kawamura *et al.*, 2010; Nikovics *et al.*, 2006). How this is mediated at the molecular level is still unclear, although some targets of the CUC transcription factors have been identified (Takeda *et al.*, 2011; Tian *et al.*, 2014).

In line with their complex roles, the specific expression patterns of *CUC* genes must be both spatially and quantitatively regulated. Their role in leaf dissection, for example, is tightly linked to expression levels: *cuc2* loss-of-function mutants show smooth leaf margins, while strongly serrated leaves are observed in *CUC2g-m4* plants that have increased *CUC2* expression levels resulting from defective regulation of *CUC2* by the microRNA miR164 (Hasson *et al.*, 2011; Nikovics *et al.*, 2006). More recently, hormonal pathways involving auxin and brassinosteroids have been identified as upstream regulators of *CUC* genes (Bilsborough *et al.*, 2011; Gendron *et al.*, 2012). In addition, several regulators, including the SWI/SNF chromatin remodelers BRAHMA and SPLAYED and the Polycomb-regulated transcriptional repressor DPA4, control the expression of *CUC* genes (Engelhorn *et al.*, 2012; Kwon *et al.*, 2006).

In order to gain further insights into the biological processes shaping plant boundaries, we designed a genetic screen to identify new components of CUC2-dependent developmental pathways. Using genetic approaches coupled with detailed morphometric analyses, we show that *MURUS1* (*MUR1*), which encodes a GDP-D-mannose 4,6-dehydratase involved in GDP-L-fucose production, is required for leaf shape development and more generally for boundary definition in plants.

## Methods

### Plant material

The *mur1-1* and *mur1-2* mutant lines originated from an ethyl methanesulfonate (EMS) screen performed in the Columbia (Col-0) genetic background and are described in Reiter *et al.* (1997). The T-DNA insertion mutant lines *cuc2-3* and *cuc3-105* in the Col-0 genetic background were reported by Hibara *et al.* (2006). The *CUC2g-m4* line was first described by Nikovics *et al.* (2006). For the genetic screen, we used a double homozygous *CUC2g-m4* line containing the *pCUC2::GUS* reporter, also described by Nikovics *et al.* (2006).

The reporter line *pCUC2::CUC2-VENUS* in the *cuc2-3* mutant background is described in Gonçalves *et al.* (2015). The *pCUC2::CUC2-VENUS cuc2-3* line was crossed into the *mur1-1* mutant and plants were taken to generation F3 to produce a line homozygous for the *pCUC2::CUC2-VENUS* reporter in a double *cuc2-3 mur1-1* homozygous mutant background.

A *pCUC2::RFP* reporter line was generated as follows: the N-terminal endoplasmic reticulum (ER)-targeting sequence of the pumpkin 2S albumin and the C-terminal ER retention signal HDEL (Matsushima *et al.*, 2002; Zhong *et al.*, 2008) were added by multiple rounds of PCR to the mRFP (monomeric red fluorescent protein) from pH7WGR2 (Karimi *et al.*, 2007). After cloning into pGEM-T (Promega), the resulting ermRFP was cloned as a *Not*I fragment behind the 3.7 kb CUC2 promoter from the pGreen0129-t35S-ProCUC2 binary vector (Hasson *et al.*, 2011). The resulting construct was transferred into *Agrobacterium tumefaciens* strain GV3101 and wild-type Col-0 Arabidopsis plants were transformed by floral dipping. Primary transformants were selected *in vitro* for their resistance to hygromycin. The expression pattern of the *pCUC2::RFP* reporter was checked in the inflorescence meristem and leaves in multiple independent lines, and one line was selected for further analysis based on the quality of the fluorescence (level, pattern, and consistency between siblings) and a segregation indicating the integration of the transgene at a single locus. The *pCUC2::RFP* reporter was introduced into the *mur1-2* mutant background by crossing and plants were taken to generation F3 to generate a line homozygous for the *pCUC2::RFP* reporter in an *mur1-2* homozygous mutant background.

To generate the *pMUR1::GUS* reporter, we amplified a 2411 bp fragment upstream of the *MUR1* coding sequence start site using the primers 5ʹ-*AAGCTT*CCCACTAGAAAAGTGACACAGT-3ʹ and 5ʹ-*CCATGG*TTGGGTTTTCGGATCTGGGA-3ʹ to introduce *Hin*dIII and *Nco*I restriction sites. The PCR product was cloned into pGEM-T (Promega). The promoter fragment was cloned into the pCAMBIA3301 binary vector using the *Hin*dIII/*Nco*I restriction site. The resulting construct was sequence verified and transferred into *Agrobacterium tumefaciens* strain GV3101, and Col-0 plants were transformed by floral dipping. Transformant plants were selected on kanamycin.

### Growth conditions

M2 and M3 plants in the genetic screen were grown in a greenhouse under long-day conditions (16 h light at 23 °C and 8 h dark at 15 °C). Plants used in leaf development and morphological studies were grown in controlled-environment rooms in either short-day [1 h dawn (19 °C, 80 µmol m^–2^ s^–1^ light), 6 h day (21 °C, 120 µmol m^–2^ s^–1^ light), 1 h dusk (20 °C, 80 µmol m^–2^ s^–1^ light), 16 h dark (18 °C, no light)] or long-day [1 h dawn (19 °C, 80 µmol m^–2^ s^–1^ light), 14 h light (21 °C, 120 µmol m^–2^ s^–1^ light), 1 h dusk (20 °C, 80 µmol m^–2^ s^–1^ light) and 8 h dark (18 °C, no light)] conditions. For *in vitro* assays, plants were grown axenically in Arabidopsis growth medium (modified from Estelle and Somerville, 1987) containing 5 mM KNO_3_, 2.5 mM KH_2_PO_4_, 2 mM MgSO_4_, 2 mM Ca(NO_3_)_2_, 70 µM H_3_BO_3_, 14 µM MnCl_2_, 0.5 µM CuSO_4_, 1 µM ZnSO_4_, 0.2 µM NaMoO_4_, 10 µM NaCl, 0.01 µM CoCl_2_, 0.005% (w/v) ammoniacal iron (III) citrate, 3.5 mM 2-(N-morpholino)ethanesulfonic acid (MES), 1% (w/v) saccharose, 1 mg l^–1^ calcium panthotenate, 0.01 mg l^–1^ biotin, 1 mg l^–1^ niacin, 1 mg l^–1^ pyridoxine, 1 mg l^–1^ thiamine, 100 mg l^–1^ inositol, 8 mg l^–1^ bromocresol purple, and 6 g l^–1^ agar, pH5.6. Plants were grown in a growth chamber under long-day conditions (16 h light/8 h dark) at 21 °C. For the L-fucose treatment, L-fucose (F2252, Sigma) was added to the medium at a final concentration of 10 mM and plants were grown in the same day length conditions.

All other phenotyping experiments were performed on plants grown in a greenhouse under long-day conditions (16 h light at 23 °C and 8 h dark at 15 °C).

### Mutagenesis, genetic suppressor screen, and sequencing

We performed EMS mutagenesis on a *CUC2g-m4* line. Seeds were mutagenized by agitation with 0.3% EMS for 17 hours, neutralized with 5 ml of 1 M sodium thiosulfate for 5 min, and washed in distilled water. Approximately 6000 mutagenized seeds were sown on soil and M2 seeds were harvested in bulks of 25 plants. Approximately 300 M2 plants per bulk were sown and screened for suppression of the leaf lobbing phenotype by visual inspection. An F2 mapping population was generated by backcrossing *folivora* to the parental *CUC2g-m4* line. Genomic DNA was isolated from ~125 *folivora* mutants segregating within the F2 and pool sequenced using Illumina technology by the Earlham Institute (formerly The Genome Analysis Centre, Norwich, UK). Sequencing data were analyzed using the SHORE and SHOREmap pipelines for identification of single nucleotide polymorphisms with high frequency within the sequenced *folivora* population (Schneeberger *et al.*, 2009).

### Xyloglucan structural analysis

Xyloglucan analysis was based on the rapid phenotyping method using enzymatic oligosaccharide fingerprinting previously described (Lerouxel *et al.*, 2002; Sechet *et al.*, 2016). Mature rosette leaves were harvested and cleared in ethanol overnight at room temperature. After removal of ethanol and rehydration in water, xyloglucan oligosaccharides were generated by digesting the samples with endoglucanase in 10 mM sodium acetate buffer, pH 5, in a final volume of 20 µl, overnight at 37 °C. An aliquot of 0.5 µl of the supernatant was dried on a matrix-assisted laser desorption/ionization time-of-flight mass (MALDI-TOF) target, followed by the addition of 0.5 µl of super-DHB (9:1 mixture of 2,5-dihydroxy-benzoic acid and 2-hydroxy-5-methoxy-benzoic acid, Sigma-Aldrich) matrix, and dried under a fume hood prior to spectra acquisition. The MALDI-TOF mass spectra of the xyloglucan oligosaccharides were acquired with a MALDI/TOF Bruker Reflex III.

### Leaf development and morphological studies

#### Sample preparation and scanning

Leaf silhouettes were prepared by sticking detached leaves on to a sheet of paper and scanning them using a Perfection V500 Photo scanner (Epson). For the developmental series, L6 leaves were dissected from the meristem under a stereomicroscope, mounted in water between a slide and coverslip, and imaged using an Axio Zoom.V16 microscope (Carl Zeiss Microscopy, Jena, Germany; http://www.zeiss.com/). For leaf shape analysis of *in vitro* grown plants, L5 leaves were collected, mounted in water between a slide and coverslip, and imaged using a stereomicroscope (SMZ 1000, Nikon) coupled to a camera (ProgResC10plus, Jenoptik).

#### MorphoLeaf

Leaf silhouettes and measurements were obtained using MorphoLeaf software, which segments leaves and extracts relevant biological features (Biot *et al.*, 2016). Output data analysis, statistics, and plots, both here and in subsequent experiments, were performed using R software (R Core Team, 2016) and the graphics package ggplot2.

#### Dissection index calculation

The leaf dissection index (DI) shape quantification method is inspired by work reported in Sicard *et al.* (2014). DI was calculated as leaf_DI=(leaf_perimeter^2^)/(4π*leaf_area) for each individual leaf. To take into account overall leaf shape, the same calculation was performed for the alpha-hull of each leaf, which is a generalization of the convex hull around the leaf serrations and petiole. Alpha-hull perimeter and area were obtained using the R package alphahull (https://CRAN.R-project.org/package=alphahull). The alpha-hull DI was calculated using the formula ahull_DI=(ahull_perimeter^2^)/(4π*ahull_area). The final DI value, which was compared between genotypes, was calculated as the ratio between leaf_DI and ahull_DI and represents an integrative value to quantify leaf shape. The R script for the calculation of DI is available on request.

### Expression data and quantification

#### Laser assisted microdissection and RNA extraction

Leaf margins were microdissected with the ZEISS PALM MicroBeam using the Fluar 5x/0.25 M27 objective. Leaves under 1 mm long were hand-dissected and placed on MMI membrane slides, and microdissected samples were collected in ZEISS AdhesiveCaps. Cutting parameters were adjusted throughout the experiment. Approximately 20 microdissected leaf margins were collected in each sample. RNA was extracted from samples using the Arcturus PicoPure RNA Isolation Kit, following the manufacturer’s instructions. RNA quality was controlled using the Agilent RNA 6000 Pico Kit.

#### Quantitative PCR analysis

Quantitative PCR analyses were performed on a Bio-Rad CFX connect machine using the SsoAdvance Universal SYBR Green Supermix following the manufacturer’s instructions. PCR conditions were as follows: 95 °C for 3 min, followed by 45 cycles of 95 °C for 10s, 63 °C for 10s, and 72 °C for 10s. Primers used for real-time PCR analysis are described in Supplementary Table S1 at *JXB* online.

#### Imaging

The *pCUC2::CUC2-VENUS* reporter line was imaged with a Leica SP5 inverted microscope (Leica Microsystems, Wetzlar, Germany; http://www.leica-microsystems.com/). Samples were excited using a 514 nm laser and fluorescence was collected with a hybrid detector at between 530 and 580 nm. The *pCUC2::RFP* reporter line was imaged using an Axio Zoom.V16 microscope (Carl Zeiss Microscopy), using a custom-made filter block (excitation band pass filter 560/25, beam splitter 585, emission band pass filter 615/24, AHF, Tübingen, Germany; https://www.ahf.de/).

#### Image analysis

*pCUC2::CUC2-VENUS* signal quantification was performed manually using ImageJ. For each leaf, fluorescence was quantified in the distal sinuses of the first pair of teeth. The CUC2-VENUS levels shown in the Results are the mean intensity of the 12 most intense nuclei in each sinus, with intensity being measured on the medial plane of each nucleus.

To identify the sinus region showing *pCUC2::RFP* expression and quantify the fluorescence signal, we developed a dedicated macro (Qpixies) on ImageJ. This macro allows the semi-automatic identification of a zone where fluorescence signal levels are above the local background levels. By automatically defining a local background centered around the zone to quantify, this macro minimizes the variability of the quantification that may result from user definition bias. Further details of the Qpixies ImageJ macro are provided in Supplementary Protocol S1.

## Results and discussion

### *CUC2g-m4* suppressor screen

**Fig. 1.**
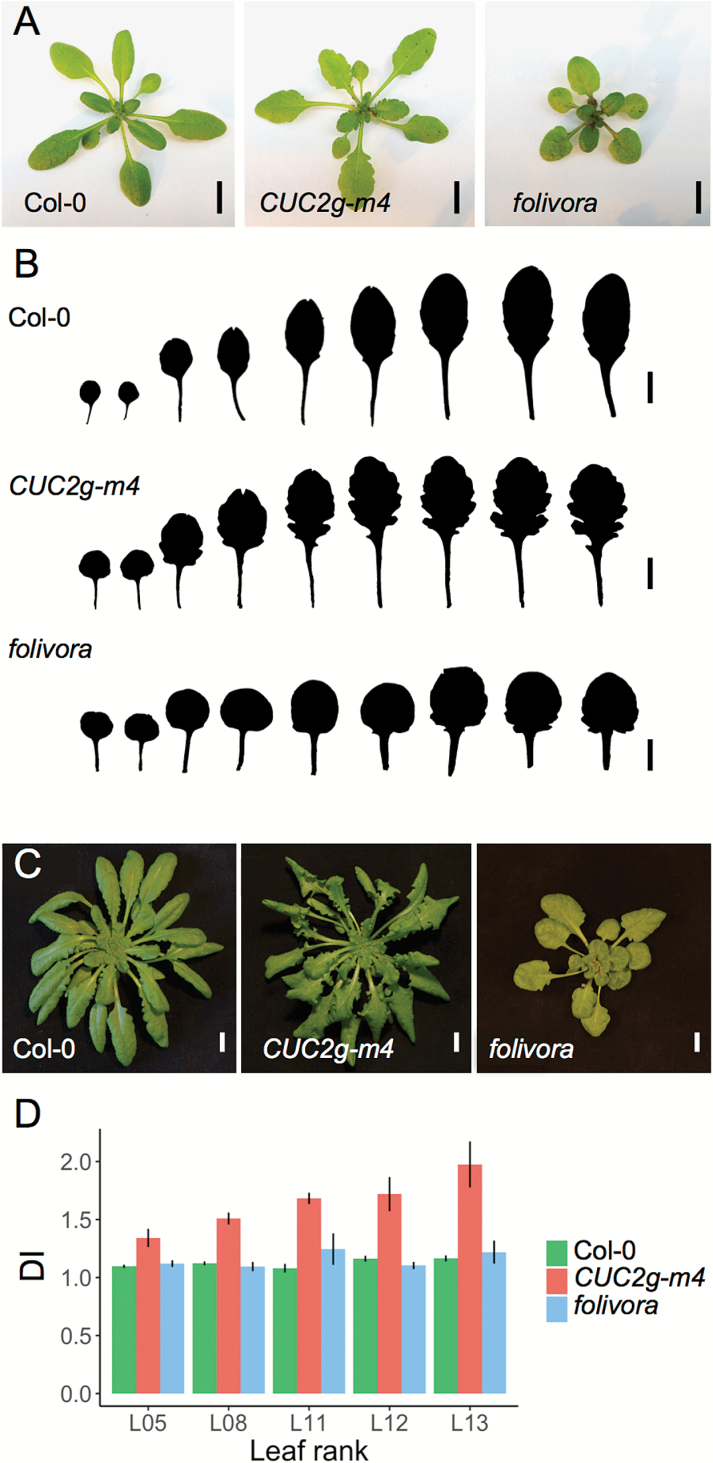
Leaf phenotypes of *CUC2g-m4* and the suppressor *folivora*. (A) Rosettes of 3-week-old *Col-0*, *CUC2g-m4*, and *folivora* plants grown in long-day conditions. (B) Leaf silhouettes of L1–L9 leaves of 4-week-old Col-0, *CUC2g-m4*, and *folivora* plants grown in long-day conditions. (C) Rosettes of 8-week-old Col-0, *CUC2g-m4*, and *folivora* plants grown in short-day conditions. (D) Mean dissection index (DI) of L5, L8, L11, L12, and L13 leaves of 8-week-day-old Col-0 (n=4 for each rank), *CUC2g-m4* (L5, n=5; L8, n=5; L11, n=4; L12, n=6; L13, n=5), and *folivora* (n=6 for each leaf rank) plants grown in short-day conditions. Error bars represent SD. Scale bars=1 cm.

To identify new regulators involved in CUC2-dependent boundary establishment pathways, we performed a genetic suppressor screen of the *CUC2g-m4* strongly dissected leaves (Fig. 1A, B) (Nikovics *et al.*, 2006). Among the M2 plants obtained after EMS mutagenesis, we isolated a mutant with short stature, rounded leaves, and reduced leaf serrations compared with the original *CUC2g-m4* line grown under long-day conditions (Fig. 1A, B). We named this mutant *folivora*, the Latin name for sloth, a mammal from the polyphyletic Edentata order (*edentata* meaning ‘toothless’). To test whether the *folivora* leaf phenotype depends on the growth conditions, we quantified the dissection level of leaves L5, L8, L11, L12, and L13 from wild type (Col-0), *CUC2g-m4*, and *folivora* plants grown in short-day conditions, using the DI as a shape descriptor. For all leaves analyzed, the DI in the *folivora* mutant was lower than that in the *CUC2g-m4* line and similar to that observed in the wild type (Fig. 1C, D). This finding indicates that EMS-induced mutation(s) in *folivora* can suppress the increase in leaf dissection associated with the *CUC2g-m4* transgene, regardless of the day length, highlighting the robustness of the *folivora* leaf phenotype.

### A mutation in *MUR1* is responsible for *CUC2g-m4* leaf phenotype suppression in *folivora*

**Fig. 2.**
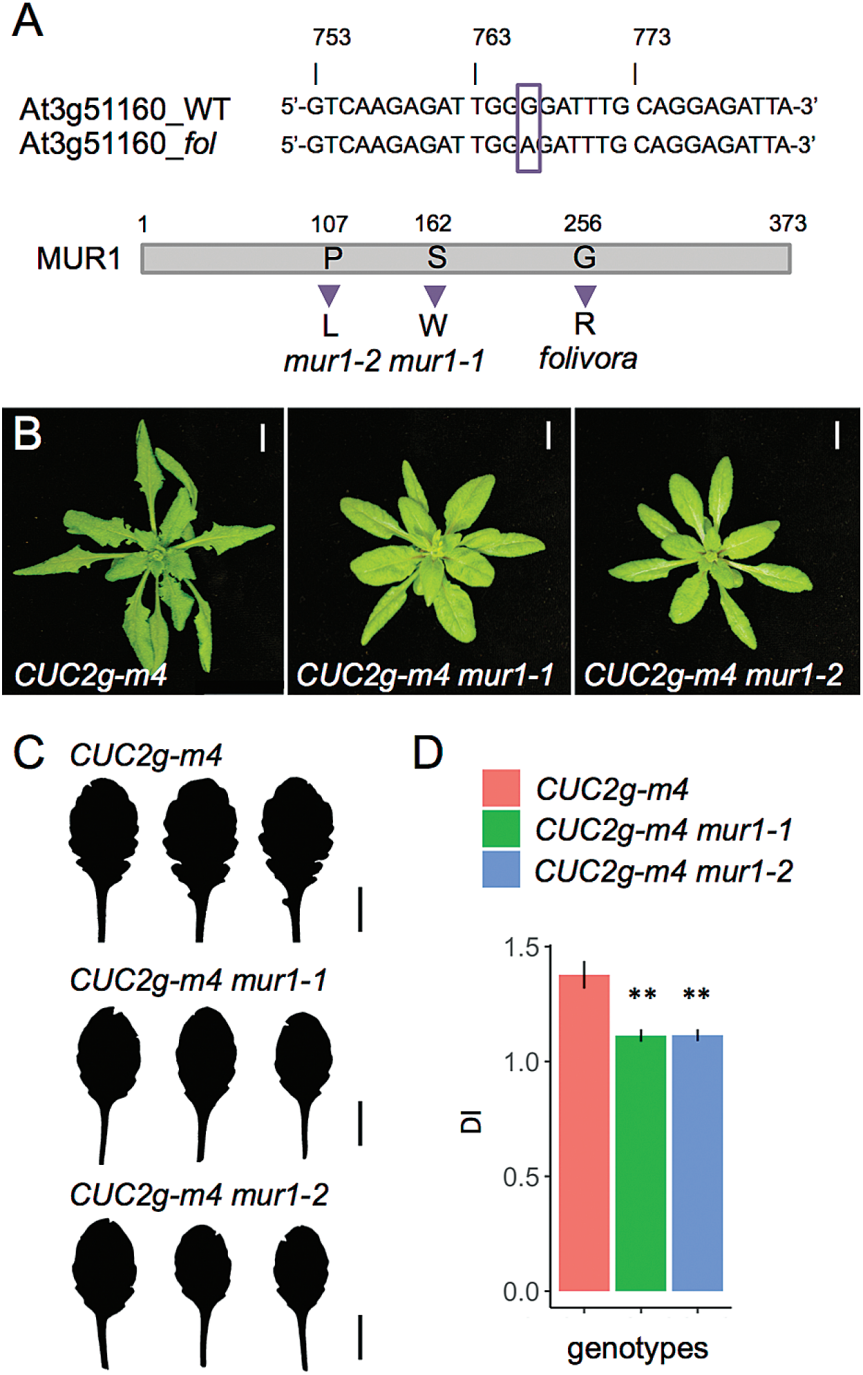
Mutations in *MUR1* suppress the *CUC2g-m4* leaf phenotype. (A) Partial genomic sequence of *MUR1* locus in the wild-type (Col-0) and the *folivora* mutant showing the G-to-A EMS-induced mutation (indicated by the box), and the predicted MUR1 amino acid sequence in the *mur1-1*, *mur1-2*, and *folivora* mutants. (B) Rosettes of 4-week-old *CUC2g-m4*, *CUC2g-m4 mur1-1*, and *CUC2g-m4 mur1-2* plants grown in long-day conditions. (C) Leaf silhouettes of three representative L6 leaves from 25-day-old *CUC2g-m4*, *CUC2g-m4 mur1-1*, and *CUC2g-m4 mur1-2* plants grown in long-day conditions. (D) Mean dissection index (DI) of L6 leaves from 25-day-old *CUC2g-m4* (n=12), *CUC2g-m4 mur1-1* (n=14), and *CUC2g-m4 mur1-2* (n=10) plants grown in long-day conditions. Error bars represent SD. **P<0.001 (Student’s *t*-test). Scale bars=1 cm.

To identify the causal mutation that suppresses the *CUC2g-m4* leaf phenotype in *folivora*, we performed backcross bulk segregant analysis using the SHORE and SHOREmap pipeline (Schneeberger *et al.*, 2009). We found an EMS-induced G-to-A mutation in the coding sequence of the locus *At3g51160* leading to a G-to-R substitution at position 256 of the MURUS1 (MUR1) protein (Fig. 2A). MUR1 is a GDP-D-mannose 4,6-dehydratase involved in GDP-L-fucose production (Bonin *et al.*, 1997). GDP-L-fucose is the activated form of L-fucose that is incorporated into cell wall glycoconjugates such as xyloglucans, rhamnogalacturonan II, and arabinogalactans (O’Neill *et al.*, 2001; Rayon *et al.*, 1999; Van Hengel and Roberts, 2002), and is involved in post-translational protein glycosylation (Strasser, 2016). *MUR1* is expressed in young leaves and in most aerial parts of the plant, with some cell type specificity (Bonin *et al.*, 2003; Supplementary Fig. S1). Expression data as well as analysis of loss-of-function mutants revealed that *MUR1* is the major gene responsible for *de novo* production of GDP-L-fucose (Reiter *et al.*, 1993; Bonin *et al.*, 2003).

The severely GDP-L-fucose-deficient *mur1-1* mutant lacks fucosylated xyloglucans, instead harboring terminal α-L-galactosyl residues (Reiter *et al.*, 1993; Zablackis *et al.*, 1996). To show that the identified mutation in *folivora MUR1* affects its encoded protein, we performed structural analysis of xyloglucans in *folivora* and compared the results to those for the previously described *mur1-1*, the wild-type, and the *CUC2g-m4* line. Structural analysis of *folivora* xyloglucans revealed a dramatic decrease in fucosylated xyloglucans (XXFG/XLFG) and the presence of α-L-galactosyl residues (XLLG), mimicking the xyloglucan structure of *mur1-1* (Supplementary Fig. S2A). We also performed allelism tests by crossing *folivora* to *mur1-1*, which bears a mutant allele of *MUR1* (Reiter *et al.*, 1993). The resulting F1 plants from *mur1-1* x *folivora* crosses had smaller leaves than the control F1 Col-0 x *folivora*, a phenotype reminiscent of the homozygous *mur1-1* mutant (Supplementary Fig. S2B; Reiter *et al.*, 1993). Together, the analysis of xyloglucan structure and our genetic analysis suggest that *folivora* harbors a hypomorphic allele of *MUR1*.

Next, to test whether the inactivation of *MUR1* is the genetic basis of the reduced leaf dissection in the *folivora* line, we introduced *mur1* mutant alleles into the *CUC2g-m4* background. The leaf shape of *CUC2g-m4* plants carrying either homozygous *mur1-1* or *mur1-2* alleles was smoother than that of *CUC2g-m4*, as shown by both L6 leaf silhouettes and DI quantification (Fig. 2B–D). Furthermore, F1 plants from the *mur1-1* x *folivora* and *mur1-2* x *folivora* crosses had smaller and smoother leaves compared with Col-0 x *folivora* F1 plants (Supplementary Fig. S2C). Altogether, these results show that *folivora* carries an inactive form of *MUR1* that suppresses the increased dissection of *CUC2g-m4* leaves, suggesting a role for GDP-L-fucose in CUC2-dependent leaf margin morphogenesis.

### Lack of GDP-L-fucose in *mur1* loss-of-function mutants leads to leaf serration defects

**Fig. 3.**
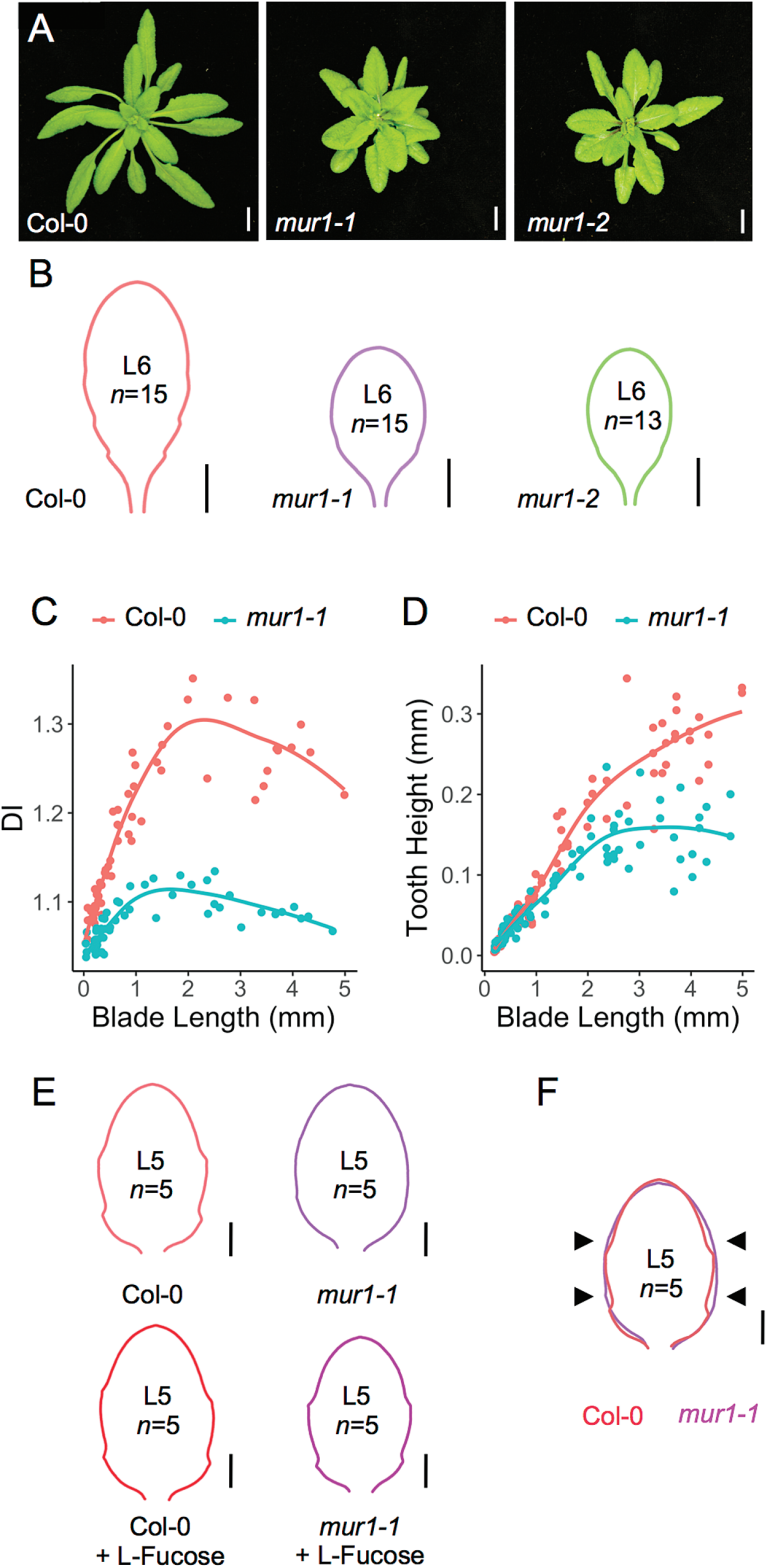
*mur1* mutants lacking GDP-L-fucose have less pronounced leaf serrations. (A) Rosettes of 4-week-old Col-0, *mur1*-*1*, and *mur1*-*2* plants grown in long-day conditions. Scale bars=1 cm. (B) Mean silhouettes of L6 leaves from 25-day-old Col-0 (n=15), *mur1-1* (n=15), and *mur1-2* (n=13) plants grown in long-day conditions. Scale bars=5 mm. (C) Dissection index (DI) plotted against blade length of L6 leaves from Col-0 (red, n=61) and *mur1*-*1* (blue, n=55) plants grown in long-day conditions. Each leaf is represented by a point, and a LOESS (local regression) curve is shown for each genotype to aid visual interpretation. (D) Tooth height of L6 first tooth plotted against blade length from Col-0 (red, n=109) and *mur1*-*1* (blue, n=99) plants grown in long-day conditions. Each tooth is represented by a point, and a LOESS curve is shown for each genotype to aid visual interpretation. (E) Mean silhouettes of L5 leaves from 23-day-old Col-0 (n=5) and *mur1*-*1* (n=5) plants grown *in vitro* in long-day conditions with and without 10 mM L-fucose supplementation. Scale bars=1 mm. (F) Superimposition of mean silhouettes of L5 leaves from Col-0 and *mur1*-*1* shown in (E), showing altered sinus formation in the *mur1-1* mutant (arrowheads). Scale bars=1 mm.

To dissect the role of *MUR1* in leaf development, we performed a detailed morphometric analysis of *mur1* loss-of-function mutants. *mur1-1* and *mur1-2* plants have smaller rosettes and leaves that appear smoother than wild-type leaves (Fig. 3A). The mature leaf shape of 25-day-old L6 leaves from Col-0, *mur1-1*, and *mur1-2* was analyzed using MorphoLeaf software (Biot *et al.*, 2016) (Fig. 3B). Mean leaf shape analysis showed that both alleles of *mur1* mutants produce leaves that are smaller and less serrated than the wild type, suggesting that *MUR1* is involved in leaf development.

Next, we reconstructed detailed developmental trajectories of leaves using MorphoLeaf (Biot *et al.*, 2016). Because the primordia initiation rate is comparable between *mur1-1* and Col-0 (Supplementary Fig. S3A) and because L6 leaf growth rates are comparable for these two genotypes up to 5 mm length (Supplementary Fig. S3B), we chose to limit our morphometric analysis to this early stage and use leaf blade length as a proxy for leaf developmental stage. Plotting L6 DI evolution against blade length revealed that leaf primordia of *mur1-1* are less dissected than the wild type, with clear shape differences appearing at an early developmental stage when leaves are <1 mm in length (Fig. 3C). To trace back the global difference in leaf shape revealed by the DI to changes in individual tooth shape, we plotted the first tooth height against blade length. This showed that *mur1-1* tooth height is reduced compared with the wild type (Fig. 3D), leading to less dissected leaves. Together, these results suggest that MUR1 activity is required for the development of leaf serrations.

To further confirm the requirement of GDP-L-fucose for leaf serration development, we supplemented *mur1-1* plants with exogenous L-fucose. GDP-L-fucose can be synthesized *de novo* via MUR1 or through a salvage pathway that converts free L-fucose via a bifunctional enzyme with L-fucokinase and GDP-L-fucose pyrophosphorylase activities (Kotake *et al.*, 2008). Previous studies have shown that exogenous L-fucose applications can rescue *mur1* growth defects and that exogenous L-fucose can be incorporated into cell walls, suggesting that the salvage pathway acts independently of MUR1 (Bonin *et al.*, 1997; Reiter *et al.*, 1993). In our study, *mur1-1* plants grown *in vitro* in standard growth medium had smoother leaves than the wild type, while *mur1-1* plants grown in medium supplemented with 10 mM L-fucose developed leaf serrations like the wild type (Fig. 3E and Supplementary Fig. 4); these results show that L-fucose itself is required for proper leaf serration in *mur1* mutants, which are deficient in GDP-L-fucose biosynthesis. When L-fucose is provided, serrations can develop in the absence of a functional MUR1 protein, showing that MUR1 protein is not required for leaf serration. Interestingly, *mur1-1* and Col-0 plants grown *in vitro* had comparable leaf sizes, showing that the leaf margin defect in the mutant does not result from the general growth defects seen in *mur1* mutants in different growth conditions (Fig. 3E).

Leaf serrations are formed by alternating teeth and sinuses along the leaf margin. Superimposing mean leaf shapes from wild-type and *mur1-1* mutant plants revealed that the *mur1* leaf outline encompasses most of the wild-type leaf outline (Fig. 3F), suggesting that GDP-L-fucose deficiency affects sinus formation.

### *CUC2* expression is reduced in *mur1* mutants

**Fig. 4.**
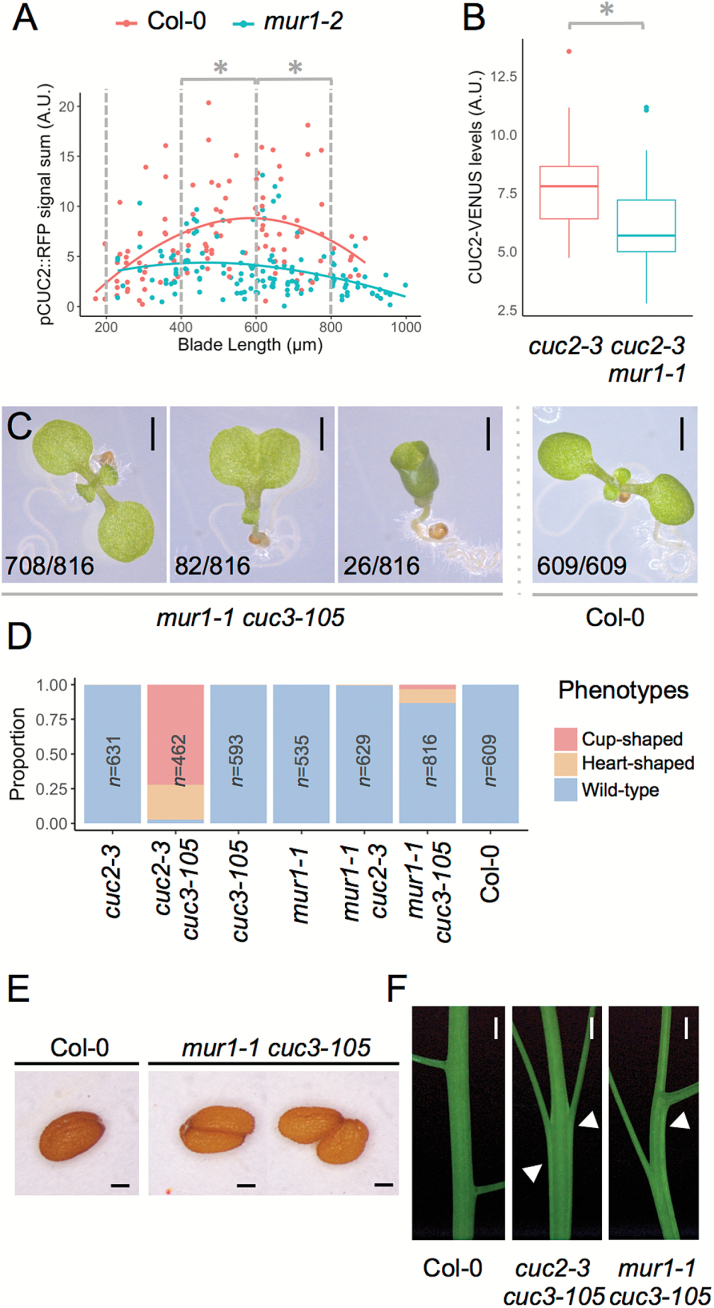
Boundary defects in GDP-L-fucose deficient mutants. (A) Quantification of CUC2 promoter activity using a *pCUC2::RFP* transcriptional reporter in the wild-type and *mur1-2* genetic background. Each point represents the accumulated signal intensity in the distal sinus of the first tooth of L6 leaves for Col-0 and *mur1-2* plants grown in long-day conditions (see Materials and methods and Supplementary Fig. S3 for details), and a LOESS (local regression) curve is shown for each genotype to aid visual interpretation. Statistical significance was calculated on three blade length classes [200–400µm, Col-0 (n=35) and *mur1-2* (n=22); 400–600 µm, Col-0 (n=30) and *mur1-2* (n=38); 600–800 µm, Col-0 (n=37) and *mur1-2* (n=42)]. *P<0.005 (Student’s *t*-test). (B) Quantification of CUC2 protein levels using the *pCUC2::CUC2-VENUS* translational fusion reporter in Col-0 (n=22) and *mur1-1* (n=38). Leaves with blade length 250–500 µm were considered. *P<0.005 (Student’s *t*-test). (C) Cotyledon fusion defects in the *cuc3-105 mur1-1* double mutant. From left to right: 9-day-old seedling grown *in vitro* with no cotyledon fusion (wild-type like); heart-shaped cotyledons; and cup-shaped cotyledons. A wild-type plant is shown in the rightmost panel. The numbers of seedlings showing each of these cotyledon phenotypes within the seedling population observed is indicated in the lower right corner of each image. Scale bars=1 mm. (D) Frequencies of cotyledon fusion defects in single and double mutants. (E) Wild-type Col-0 seed (left panel) and seed fusion defects in the *cuc3-105 mur1-1* double mutant (right panel). Scale bars=100 µm. (F) Stem fusion (fasciation; arrowheads) defects in the *cuc3-105 mur1-1* and *cuc2-3 cuc3-105* double mutants, compared with a wild-type (Col-0) stem. Scale bars=1 mm.

CUC2 is an important regulator of leaf serration development (Biot *et al.*, 2016). Because GDP-L-fucose deficiency led to reduced leaf serration even in the highly serrated *CUC2gm-4* line, we hypothesized that *CUC2* expression is modified in the *mur1* background. To test this hypothesis, we used a transgenic line reporting the transcriptional activity of the CUC2 promoter (*pCUC2::RFP*). We analyzed the expression of this reporter in developing leaves of both wild-type and *mur1-2* mutant backgrounds by quantifying RFP fluorescence levels. CUC2 promoter activity was reduced in the sinuses at early developmental stages in *mur1-2* compared with the wild type for leaves between 400 and 800 µm in length (Fig. 4A), suggesting that in the absence of GDP-L-fucose CUC2 promoter activity is impaired in developing leaves. We used laser-assisted microdissection to measure relative *CUC2* mRNA accumulation in the wild-type and *mur1-1* leaf margins*. CUC2* mRNA levels were reduced in *mur1-1* compared with the wild type (Supplementary Fig. S5), confirming the results obtained with the *CUC2* transcriptional reporter.

Because *CUC2* expression is regulated post-transcriptionally by a microRNA, miR164, we next determined whether the reduced transcriptional activity of *CUC2* had an impact on CUC2 protein accumulation. For this, we measured CUC2 protein levels in *mur1-1* leaves using a translational fusion reporter *pCUC2::CUC2-VENUS* that complements the *cuc2-3* mutant allele. We quantified the CUC2-VENUS signal in distal leaf sinuses of tooth 1 and found that CUC2 protein levels are lower in the *mur1-1* mutant background (Fig. 4B). Hence, *CUC2* is expressed at lower levels in a GDP-L-fucose deficient mutant, in a manner consistent with the reduced sinus formation observed in the *mur1-1* mutant.

### GDP-L-fucose is required in CUC2-dependent boundary formation

The *CUC2* transcription factor acts in a partially redundant manner with *CUC3* to regulate several aspects of plant development that require boundary definition and tissue separation. Notably, *CUC2* and *CUC3* are required for proper separation of cotyledons, individualization of ovule primordia, and separation of the flower pedicel from the stem (Burian *et al.*, 2015; Gonçalves *et al.*, 2015; Hibara *et al.*, 2006). To investigate whether GDP-L-fucose is generally required with *CUC2* and/or *CUC3* in such different developmental contexts, we generated the *mur1-1* c*uc2-3* and *mur1-1 cuc3-105* double mutants and analyzed fusion phenotypes in cotyledons, stem, and seeds. *cuc2-3* and *cuc3-105* single mutants have low frequencies of heart-shaped cotyledon phenotypes but no cup-shaped cotyledon phenotypes (Hibara *et al.*, 2006). While *mur1-1 cuc2-3* double mutants show few to no cotyledon fusions, much like the respective single mutants, *mur1-1 cuc3-105* double mutants show strong cotyledon fusions (cup- or heart-shaped cotyledons) like those observed in *cuc2-3 cuc3-105* double mutants (Fig. 4C, D). Similarly, seed fusion and stem fasciation defects are observed in the *mur1-1 cuc3-105* double mutant but not in the *mur1-1 cuc2-3* mutant background or the single mutants (Fig. 4E, F). Because *mur1-1* specifically enhances the phenotype of the *cuc3-105* mutant and not that of *cuc2-3*, and because *mur1-1 cuc3-105* double mutants phenocopy several defects of the *cuc2-3 cuc3-105* double mutant, we propose that *MUR1* acts in the same pathway as *CUC2* to promote organ separation in different developmental contexts.

## Conclusion

Collectively, our results show that GDP-L-fucose is required for the definition of organ boundaries during plant development, and that this occurs at least partially via a pathway involving CUC2. GDP-L-fucose has several roles during plant development. For instance, the dwarf phenotype of *mur1-1* and *mur1-2* mutants has been attributed to reduced fucose incorporation into rhamnogalacturonan II (O’Neill *et al.*, 2001). Xyloglucan fucosylation is also impaired in the *mur1*, mutant which instead harbors terminal α-L-galactosyl residues (Reiter *et al.*, 1993; Zablackis *et al.*, 1996). Fucose can also be added to proteins in the ER by the activity of specific fucosyltransferases, which therefore have multiple roles depending on the fucosylated targets (Strasser, 2016). However, it is not known how GDP-L-fucose changes *CUC2* expression levels. As GDP-L-fucose is involved in post-translational modifications of proteins, one hypothesis is that the activity of one or several upstream regulators of *CUC2* expression is dependent on such modifications. Interestingly, a parallel can be drawn with *Drosophila* development, in which GDP-L-fucose deficiency affects cell-signaling mechanisms required for the proper expression of *wingless* (*wg*) at the dorsoventral boundary of imaginal wing discs. More precisely, *O*-fucosylation of the NOTCH cell-surface receptor impacts its ligand-binding capacity, thus fine-tuning NOTCH signaling and subsequent activation of *wg* expression (Ayukawa *et al.*, 2012; Kopan, 2012; Okajima *et al.*, 2005; Stanley, 2007). Another hypothesis is that *CUC2* expression is indirectly impacted by changes in cell wall properties triggered by GDP-L-fucose deficiency. Finally, independently or downstream of *CUC2* expression, such modifications of cell wall properties and/or post-translational modifications of proteins may also affect the differential growth that accompanies boundary function. Although further analyses will be necessary to pinpoint how GDP-L-fucose modulates plant boundary definition, here we provide evidence for a new role for GDP-L-fucose in leaf shape development and, more generally, boundary domain definition, and broaden our understanding of plant architecture development.

## Supplementary data

Supplementary data are available at *JXB* online.

Fig. S1. MUR1 expression pattern.

Fig. S2. *folivora* harbors a hypomorphic allele of *MUR1*.

Fig. S3. Col-0 and *mur1-1* leaf initiation and growth parameters.

Fig. S4. Original data showing that L-fucose treatment restores *mur1-1* leaf serration phenotype *in vitro*.

Fig. S5. Analysis of CUC2 expression in wild type and *mur1-1* leaf margins.

Table S1. List of qPCR primers used in this study.

Protocol S1. Qpixies macro for ImageJ.

Supplementary Material

## Competing interests

The authors declare no competing or financial interests.

## Author contributions

BG, PL, and NA conceived and designed the experiments. BG, AM-C, BA, AH, and NA performed the experiments with input from GM and PL. BG and EG performed xyloglucan structure analysis. NB and NA performed laser-assisted microdissection experiments. AH generated the *pCUC2::RFP* transgenic line. BG, MC, TB, and PL developed the Qpixies macro. BG, PL, and NA wrote the paper with input from the other authors.
