# Supplementary material for: GDP-L-fucose is required for boundary definition in plants
Source: J Exp Bot. 2017 Nov 25;68(21-22):5801–11. doi: 10.1093/jxb/erx402 (PMC5854112; doi:10.1093/jxb/erx402)
Supplement: Supplementary Material [file erx402_suppl_supplementary_material.pdf]

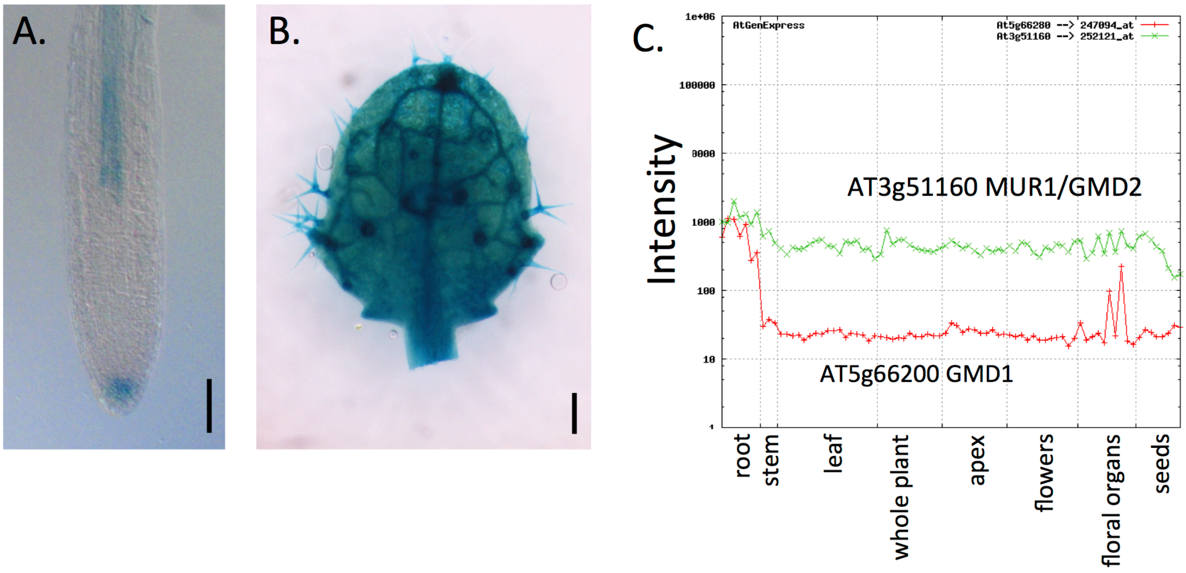

Supplementary Figure 1: ***MUR1* has a wide expression pattern throughout the plant.**

A. Primary root tip from *pMUR1::GUS* line. Scale bar=50 µm B. Developing leaf from pMUR1::GUS line. Scale bar=200µm. C. Expression data from AtGenExpress (http://jsp.weigelworld.org/expviz/expviz.jsp) showing expression levels for At3g51160 MUR1/GMD2 (green) and At5g66200 GMD1 (red) in different plant organs.

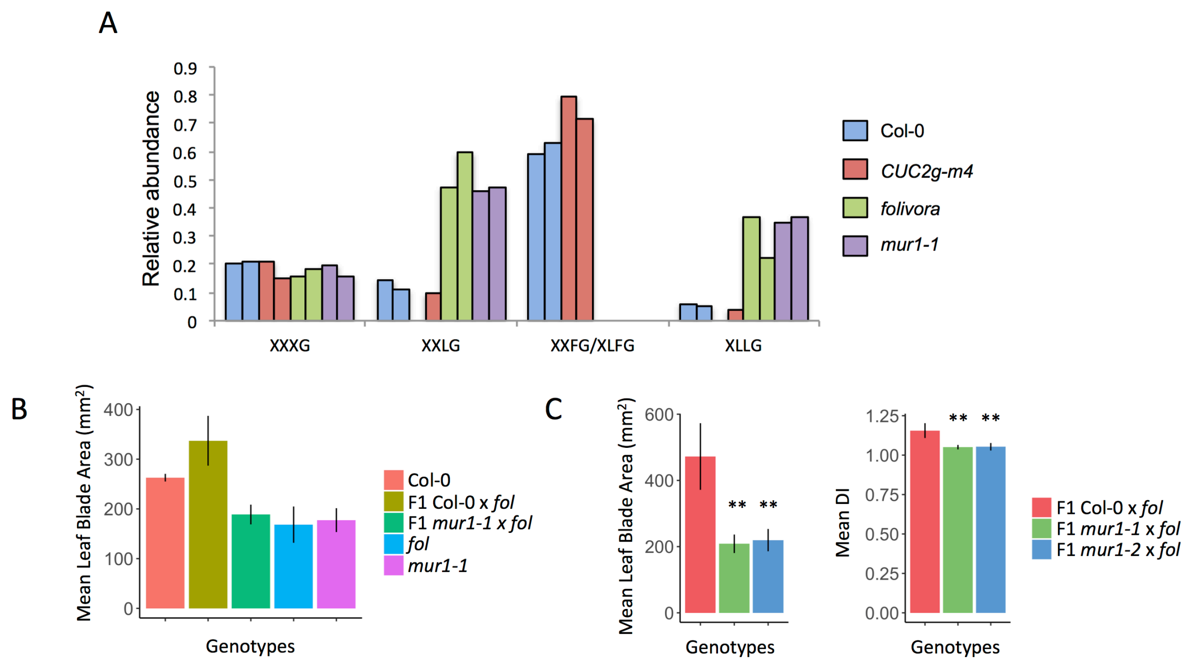

Supplementary Figure 2: *folivora* is allelic to *mur1* mutants.

A. Xyloglucans structure in 2 biological replicates from Col-0, *CUCg-m4, folivora* and *mur1-1* rosettes leaves. Acetylated oligosaccharides are included.

B. Mean Leaf 6 Blade area of Col-0, F1 plants from Col-0 x *fol* and *mur1-1* x *fol, folivora and mur1-1* mutants (n=3). Error bars represent standard deviation (SD).

C. Mean Leaf 6 Blade Area and mean Leaf 6 DI of F1 plants from Col-0 x *fol* (*n*=8), *mur1-1* x *fol* (*n*=9), *mur1-2* x *fol* (*n*=7). Error bars represent standard deviation (SD). ** pvalue<0.001

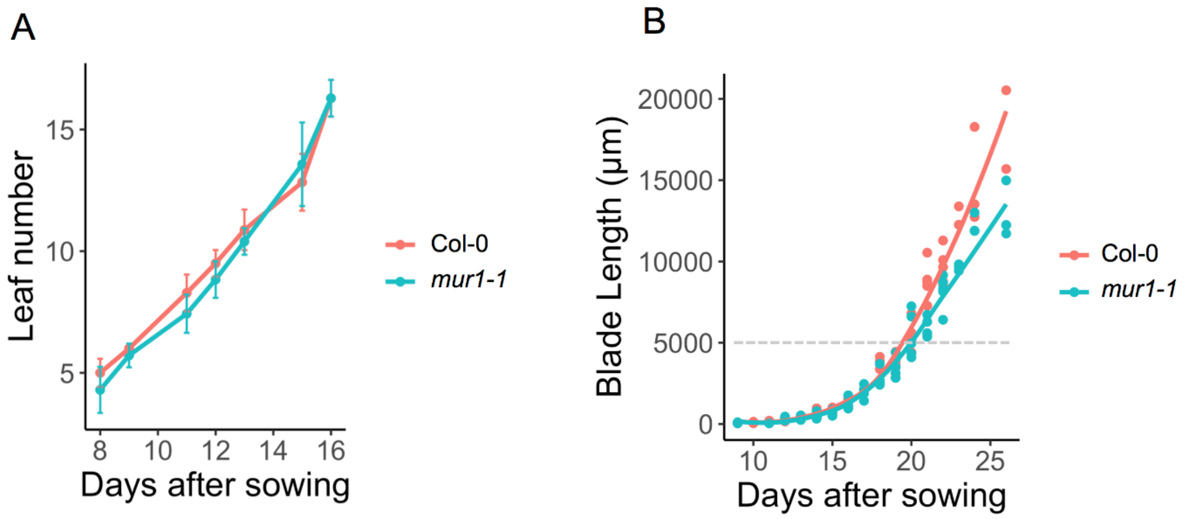

Supplementary Figure 3: Col-0 and *mur1-1* leaf initiation and growth parameters.
A. Col-0 (d8 n=7, d9 n=7, d11 n=7, d12 n=6, d13 n=8, d15 n=6, d16 n=7) and *mur1-1* (d8 n=10, d9 n=7, d11 n=7, d12 n=6, d13 n=5, d15 n=7, d16 n=7) leaf primordia appearance in long day conditions. Error bars represent SD.
B. Col-0 (red, n=83) and *mur1-1* (blue, n=78) blade length during leaf development. Each leaf is represented by a point and a LOESS (local regression) curve is shown for each genotype to aid visual interpretation.

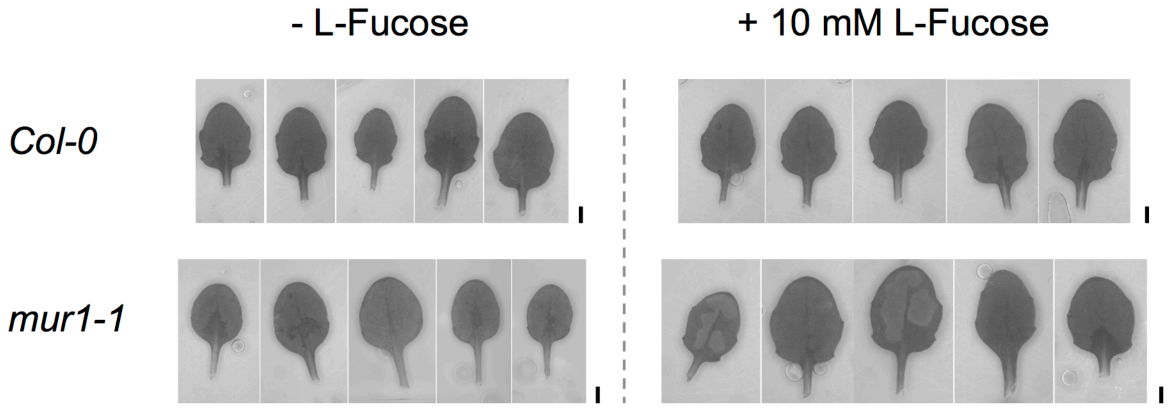

Supplementary Figure 4: L-Fucose treatment restores *mur1-1* leaf serration phenotype *in vitro*.
Images of leaves (L5) from 23-day-old Col-0 and *mur1-1* plants grown *in vitro* in long-day conditions with and without 10 mM L-fucose supplement. These images have been used to calculate the mean leaf shape represented on Figure 3 E and F. Scale bars =1 mm.

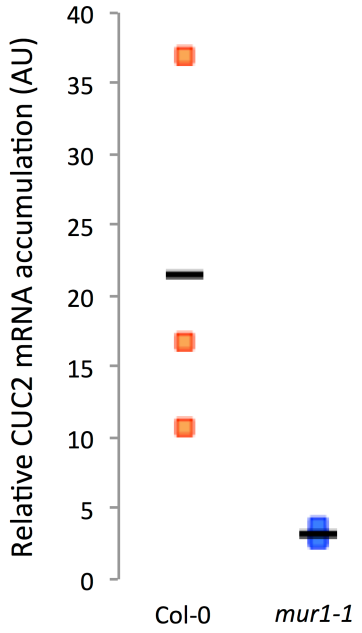

Supplementary Figure 5: Analysis of CUC2 expression in wild type and *mur1-1* leaf margins.

Total mRNA was extracted from Laser-Assisted Microdissected margins of leaves smaller 1000µm in length. *CUC2* mRNA accumulation was measured using qPCR and normalized by *qREF* (At2g28390*)*. The squares represent individual data points while the black dash represents the calculated mean value (n=3).

Supplementary Table 1: List of primers used in this study

Primers	Targets	Sequence 5'-3'	Reference
qREF-F	AT2G28390	AACTCTATGCAGCATTTGATCCACT	Morineau et al., 2016
qREF-R	AT2G28390	TGATTGCATATCTTTATCGCCATC	Morineau et al., 2016
qCUC2-F	AT5G53950	CTTGGCAACTTCCCGGGAGA	Tian et al., 2014
qCUC2-R	AT5G53950	CCAGCCTCAGTTGCTCTGTTAGTT	Tian et al., 2014

Morineau, C., Gissot, L., Bellec, Y., Hematy, K., Tellier, F., Renne, C., Haslam, R., Beaudoin, F., Napier, J., and Faure, J. D. (2016). Dual fatty acid elongase complex interactions in arabidopsis. PLoS One 11, 1–20.

Tian, C., Zhang, X., He, J., Yu, H., Wang, Y., Shi, B., Han, Y., Wang, G., Feng, X., Zhang, C., et al. (2014). An organ boundary-enriched gene regulatory network uncovers regulatory hierarchies underlying axillary meristem initiation. Molecular Systems Biology 10, 755.

Supplementary Protocol : Qpixies Macro For ImageJ

This ImageJ macro allows the semi-automatic identification of three parameters associated with a fluorescence signal: a zone with a signal level above the local background level; a zone with a user-defined area in which the signal is maximum; and a local background zone. By automatically defining a local background centred around the zone to be quantified, this macro minimizes the variability of the quantification that may result from user bias in the definition of the initial zone to be analysed. The input images can be single calibrated TIFF pictures or stacks of multiple calibrated TIFF slices corresponding to different channels and/or different exposure times.

1. Optional denoising and Manual Selection of the Zone Analysed

An optional image denoising step can be performed through the application of a Gaussian blur.

The user manually defines a large zone enclosing the region to be quantified.

ZoneAnalysed

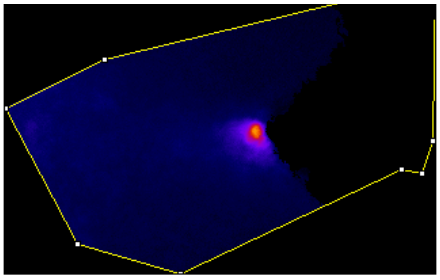

2. Definition of the Object

The Object (here a part of leaf) is distinguished from the background by thresholding the image.

Object

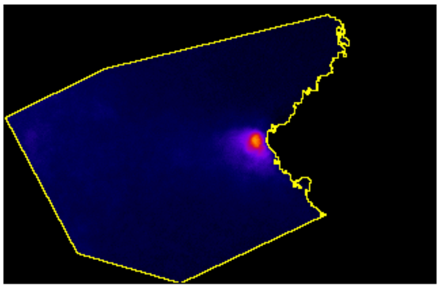

3. Definition of the BrightestArea

The BrightestArea is defined as a continuous zone in which the pixel intensity is above the mean intensity plus two times the standard deviation of the mean (mean+2SD) of the pixel intensity within the Object.

BrightestArea

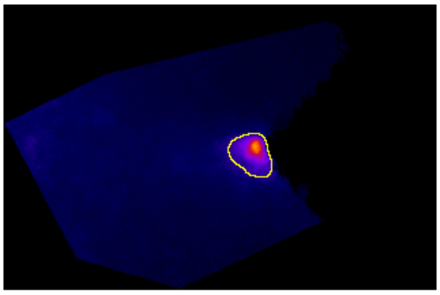

4. Definition of the BackGround

The BackGround is defined as the Object from which is excluded the Brightest Area.

BackGround

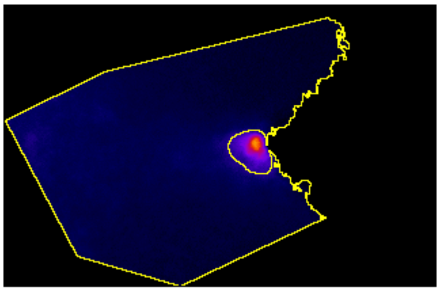

5. Refining the SignalEstimation

In step 3, the mean intensity is calculated in the Object, which includes the zone with a strong signal and the background zone. This leads to an overestimation of the pixel intensity in the background and hence an underestimation of the signal. To overcome this, the SignalEstimation is defined here as a continuous zone in which the pixel intensity is above the mean+2SD of the pixel intensity within the Background.

SignalEstimation

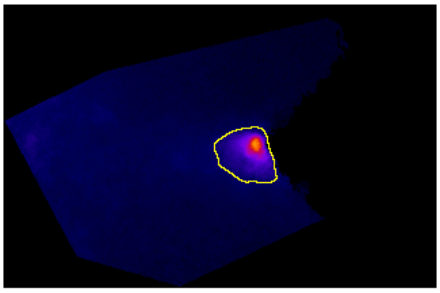

6. Definition of the TargetArea

The background is calculated in a zone that depends on the Object defined by the user in step 1. Therefore, analysis of the same image with different Objects may lead to different final results. To limits this, we introduce this step that defines the TargetArea. The TargetArea is defined as the intersection of the Object with a Disk centred on the centre of the SignalEstimation and a radius equal to the radius of SignalEstimation multiplied by a user-defined constant (here 2).

TargetArea

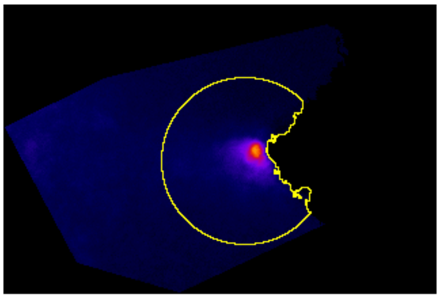

7. Definition of the LocalBackground

The LocalBackground is defined as the TargetArea from which the SignalEstimation is excluded

LocalBackground

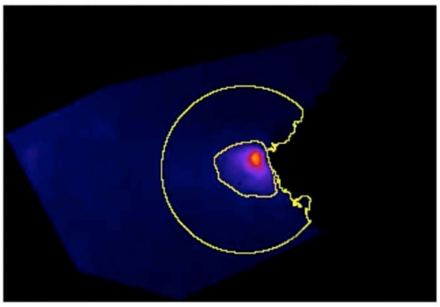

8. Definition of the RawSignal

The RawSignal is calculated taking into account the local background: it is defined as a continuous zone in which the pixel intensity is above the mean+2SD of the pixel intensity within the LocalBackground.

Raw Signal

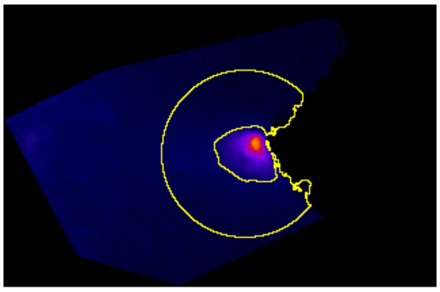

9. Definition of the Signal

To refine the Signal zone, the steps 7 and 8 are iterated 10 times, or until the change in area of the RawSignal between two iterations is <1%.

Signal

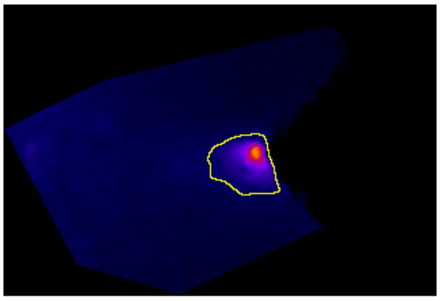

10. Definition of the SignalMaximum

The SignalMaximum is defined as continuous zone with an area defined by the user and that corresponds to the intersection of the Signal and a disk centred on the centre of the Signal.

SignalMaximum

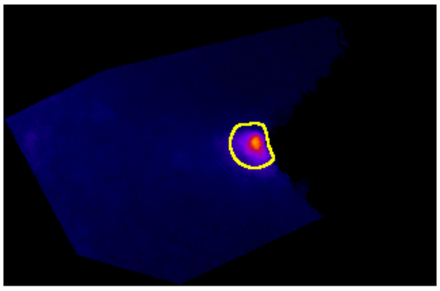

The output of the macro is the area, mean pixel intensity and SD of the three zones defined (Signal, SignalMaximum and LocalBackground). If the initial image contains multiples slices, these parameters will be measured for each zone in each slice.
